# Supplementary material for: Biologically Plausible Training Mechanisms for Self-Supervised Learning in Deep Networks
Source: Front Comput Neurosci. 2022 Mar 21;16:789253. doi: 10.3389/fncom.2022.789253 (PMC8977509; doi:10.3389/fncom.2022.789253)
Supplement: Supplementary file 1 [file Presentation_1.pdf]

Appendix: Biologically Plausible Training Mechanisms for Self-Supervised Learning in Deep Networks

Mufeng Tang, Yibo Yang
Department of Statistics
University of Chicago, Chicago, IL, USA
{mufengt,yiboyang}@uchicago.edu

Yali Amit
Department of Statistics
University of Chicago, Chicago, IL, USA
amit@marx.uchicago.edu

A Computing the thresholds for the error signal at the top layer

For each unit i in layer L one can imagine using an inhibitory unit u_i^I activated by $x_{L,i}^B$ and another unit $x_i^{D,+} = x_{L,i}^A + u_i^I$. Similarly an inhibitory unit v_i^I is activated by $x_{L,i}^A$ and $x^{D,-} = v_i^I + x_{L,i}^B$. Finally two units compute the thresholded sums: $x^{S,+} = \sum_i x_i^{D,+} > m_1, x^{S,-} = \sum_i x_i^{D,-} > m_2$, and if any of these is above threshold the quantity in $x_i^{D,+}$ is passed on as $\delta_{L,i}^B$. A similar architecture can be used to compute $\delta_{t,L,i}^B$ for each negative t.

B Examples of deformations

For the experiments with CIFAR and EMNIST/MNIST we use random affine transformations to deform the images and get positive/negative pairs. The affine transformations include rotation, scaling, shifting, as well as flipping. For CIFAR100 in particular, we also apply random color jitters that changes the saturation and hue of the images. These deformations can be used to model the variety of views of a 3D object that form the 'positive pairs' in reality. Some examples of the transformed CIFAR100 images can be found in Figure 1 (a).

There are two types of transformations used in our experiments with EMNIST/MNIST. For creating the positive pairs in training the base encoder, we apply the same affine transformation we used for CIFAR100 to EMNIST, but without the color jitters. Then for testing the perturbation invariability of our SSL method, we apply random rotation, shifting and scaling to the MNIST dataset to creat a transformed MNIST dataset. The classifier is trained on the original MNIST and tested on the transformed MNIST. Examples of the transformations applied to EMNIST/MNIST can be found in Figure 1 (b) and (c).

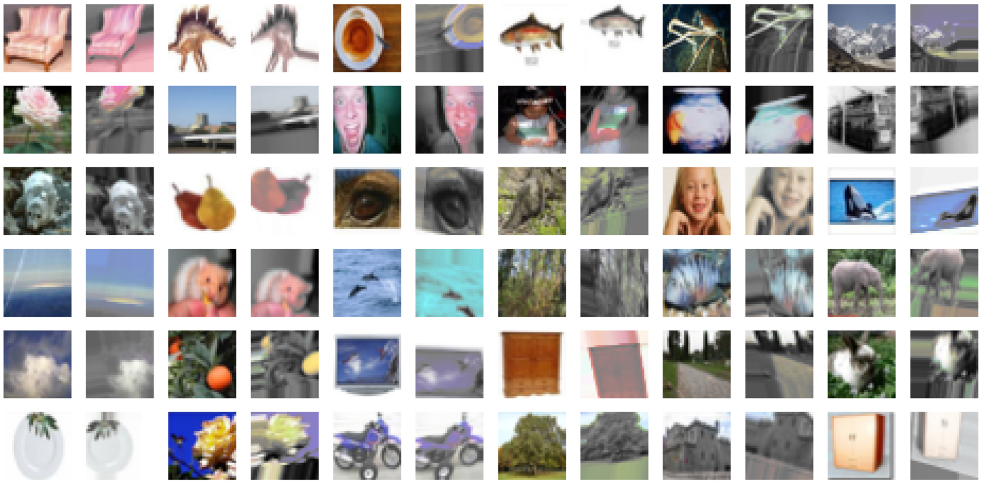

(a)

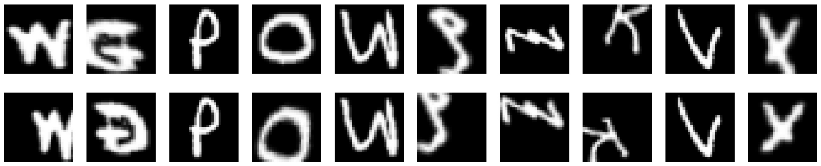

(b)

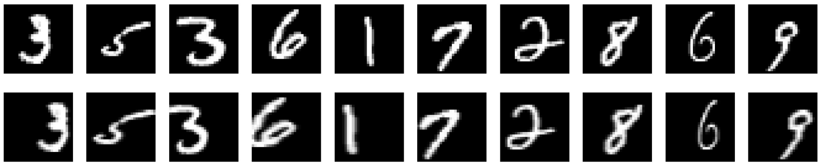

(c)

Figure 1: Examples of pairs of CIFAR100/EMNIST/MNIST images, deformed using random affine transformations and color jitters. Notice that the transformations on MNIST is slight, but have already led to performance degradation on supervised networks.

C More results with CIFAR

Results with SimCLR. We provide here the linear evaluation results with SimCLR and biologically more plausible learning rules, to provide different levels of baselines for our experiments with the contrastive hinge loss. The results are obtained with exactly the same experimental settings for contrastive hinge loss. As can be seen from Table 1 the results with SimCLR are almost identical to what we obtained with contrastive hinge loss.
Effects of head size. We run a set of preliminary experiments with end-to-end BP and the SimCLR loss to investigate whether the head size in our network will affect the performance. The results in Table 2 show that the size of the final projection head does not have a significant impact on linear evaluation performance. We thus keep the head size 64 for all our experiments:

Loss	Learning	Update	Classifier acc.
SimCLR	E2E	BP	72.44%
		RF	62.38%
		DTP	71.00%
	GLL	BP/URF	72.52%
		RF	67.19%
	RLL	BP/URF	68.99%
		RF	66.39%

Table 1: Linear evaluation results with CIFAR10, using a SimCLR-trained base encoder with CIFAR100. We compare End-to-End (E2E), Greedy Layer-wise Learning (GLL) and Randomized Layer-wise Learning (RLL), and four updating methods: back-propagation (BP), Updated Random Feedbackau (URF), Random Feedback (RF) and Difference Target Propagation (DTP)

**Head Size**	64	512	1024	2048
	69.87%	69.64%	69.97%	70.20%

Table 2: Investigation into head sizes. The linear evaluation results are obtained from a base encoder trained with CIFAR10 and classifier trained/tested on CIFAR10 as well. We use SimCLR for these experiments.

D Code

The code for our simulations and experiments can be found at:
https://github.com/C16Mftang/biological-SSL
